# Gender differences in the association of obesity-related measures with multi-morbidity among older adults in India: evidence from LASI, Wave-1

**DOI:** 10.1186/s12877-022-02869-z

**Published:** 2022-03-01

**Authors:** T. Muhammad, Bandita Boro, Manish Kumar, Shobhit Srivastava

**Affiliations:** 1 International Institute for Population Sciences, Mumbai, Maharashtra 400088 India; 2 Centre for the Study of Regional Development (CSRD), School of Social Sciences-3 (SSS 3), Jawaharlal Nehru University (JNU), New Delhi, 110067 India

**Keywords:** Obesity-related measure, Multimorbidity, Older adults, India

## Abstract

**Background:**

Co-existence of multiple chronic diseases is increasingly becoming a norm among ageing population. The study aims to investigate the prevalence of multimorbidity and the association between anthropometric measures of obesity and multimorbidity among men and women aged 60 years and above in India.

**Methods:**

The present study is based on the first wave of the Longitudinal Aging Study in India. The analytical sample size for the study was 28,050 older adults aged 60 years and above. Descriptive statistics and multivariable analysis using logistic regression models were conducted.

**Results:**

Body Mass Index (BMI) based-obesity is more prevalent among older women than men (26.3% vs. 17.6%). Similarly, higher proportion of older women was at high-risk waist circumference (37.1% vs 8.9%) and waist-hip ratio (78.5 vs 75.4%) than men respectively. In Model-I, after controlling for several covariates, older adults with overweight/obesity were 1.6 times more likely to have multi-morbidity than non-obese older adults (Adjusted OR = 1.61; 95% CI: 1.48–1.74). Similarly, older adults with high-risk waist circumference [Adjusted OR: 1.66; 95% CI: 1.52–1.80] and waist-hip ratio [Adjusted OR: 1.45; 95% CI: 1.33–1.59] also had higher odds of having multi-morbidity in reference to their counterparts. In model-3 it was found that females with high-risk waist-hip ratio had 14% lower odds of multimorbidity than males with high-risk waist-hip ratio [Adjusted OR: 0.86; 95%CI: 0.78–0.94].

**Conclusion:**

The findings of the study show significant gender difference in the prevalence of multimorbidity, men being at increased risk in the multivariate analysis which is uncommon in the existing epidemiological research. Interactive effect of male gender with anthropometric measures on multimorbidity reported in our study probably due to increased unhealthy behaviours among men requires further research.

## Background

According to the Global Burden of Disease 2015 study, higher body mass index (BMI) is one of the highest and increasing risk factor contributing to poor health conditions [1]. A study in 2013 showed that nearly one third of the world’s population was either overweight or obese [2]. More than 50% of the individuals with obesity in the world were found in 10 countries and India accounted for 15% in 2013 [3]. A higher prevalence of malnutrition, as characterized by underweight and overweight has been reported among the ageing population in India [4]. The increase in the double burden of overweight and obesity is also leading to a rise in chronic conditions which is an emerging problem in Asian countries including India [5].

Multi-morbidity at older ages leads to low quality of life, higher mortality rates, low physical and mental health, cognitive decline and higher healthcare expenditure [6, 7]. In a systematic analysis of the prevalence of multi-morbidity in high, low and middle-income countries, it was found that more than 50% of those older than 65 years had multi-morbidity and that females were affected more [8]. A couple of studies in developing countries also found that more than half of the older adults had three plus chronic conditions [9, 10]. In India, multi-morbidity is highly prevalent among older adults [11, 12], and significant gender differences have been reported in prevalence of multi-morbidity and associated mortality [12, 13]. The increase in BMI may increases the impairments in physical functioning at older ages and lead to a greater risk of developing multi-morbidity for older adults [14–17]. Previous studies revealed that prevention of overweight and obesity among older adults can help in reducing the burden of chronic diseases [18, 19]. Along with obesity, anthropometric measures such as waist circumference and waist-hip ratio also provide an indirect evaluation of body composition and are considered as associated factors of chronic diseases as they are directly linked to excess body fat [20–22]. However, studies on waist circumference and waist-hip ratio as risk factors of multi-morbidity are scarce in India.

**Fig. 1:**
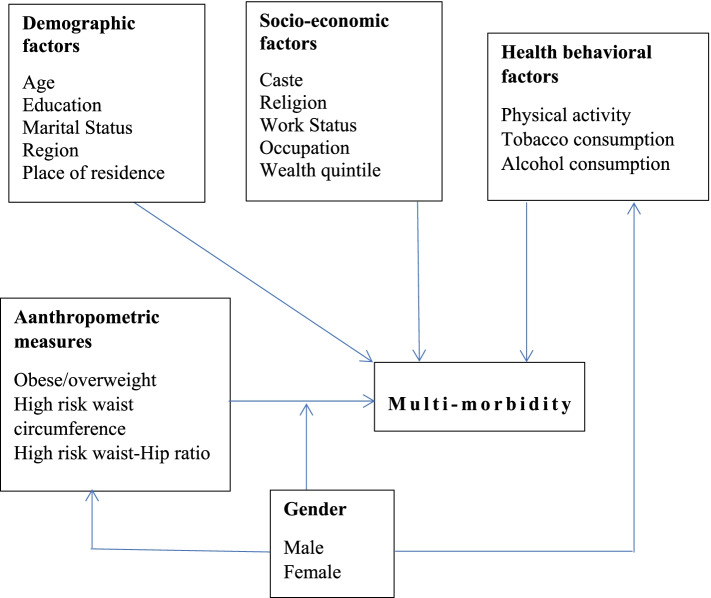
Conceptual framework of the study

Co-existence of multiple chronic diseases is increasingly becoming a norm among ageing population [23]. On the other hand, a recent study found that the prevalence of overweight and obesity among Indian adults aged 20–69 years will be tripled by 2040, and the largest increase will be seen in the older adults [24]. Therefore, a significant gender gap can be seen in the prevalence of obesity and overweight and thus it can have differential effects on multi-morbidity in older men and women. Although a significant gender difference in the prevalence of multi-morbidity was shown in a recent study in India [25], a gender-specific analysis of the association of anthropometric measures of obesity and multi-morbidity may offer insight into the differential contribution of these factors to the burden of multimorbidity in older men and women and the potential for prevention of multimorbidity in older Indian adults. This study is aimed to explore the association of obesity-related anthropometric indices with multi-morbidity in adults aged 60 years and above and the interaction of gender in those associations. Based on the abovementioned review of existing studies, a conceptual framework has been developed and summarized in Fig. 1.

## Methods

### Data

The present study is based on the first wave of the Longitudinal Aging Study in India (LASI) conducted during 2017–18 [26]. The LASI is a nationally representative longitudinal survey of middle-and older-aged adults in India (i.e., aged 45 years or older) and their spouses who reside in the same households, irrespective of age. The LASI survey provides rich information on demographics, morbidity, health behaviour factors, and physical health of the aging population in India. The major aim of the survey was to measure health and its determinants and consequences over the later stages of life. The survey adopted a multistage stratified area probability cluster sampling design. It is a nationally representative survey of 72,250 individuals aged 45 and above across all states and union territories of India. The LASI is envisioned to be conducted every two years for the next 25 years [26]. The number of targeted primary sampling units (PSUs) in a state was chosen proportionally to each sub-state area in the first step with the selection of PSUs (sub-districts or Tehsils/Talukas) (level 1 stratification). Thus, the PSUs were chosen using Probability Proportional to Size (PPS) sampling in each area, with the number of households in each PSU serving as the size measure. All PSUs (sub-districts) within each of these areas were specifically stratified using one or more of the following stratifying variables: 1) the total number of households in a sub-district, 2) the level of female literacy, 3) the proportion of Scheduled caste and Scheduled tribe population, and 4) the proportion of males employed in non-agricultural activities. The second stage entailed selecting a predetermined number of secondary sampling units (SSUs) from the selected PSUs, which are villages in rural regions and wards in urban areas. The third step in rural regions entailed selecting a set number of homes (HHs) (i.e. 32) from each designated village or village segment (for villages with more than 500 HHs). In metropolitan regions, the fourth round of selection entailed selecting a set number of HHs (35 in this case) from each census enumeration block (CEB). The interviews were conducted using computer-assisted personal interview (CAPI).

The LASI survey is conceptually comparable to the United States Health and Retirement Study (HRS) and other HRS-type surveys in various countries, including China (China Health and Retirement Longitudinal Survey) and England (English Longitudinal Study of Ageing). Along with its uniqueness of comparability with studies in other countries, LASI also considered features unique to India, including its institutional and cultural characteristics. LASI is conducted through a partnership of the International Institute of Population Sciences (IIPS), Harvard University, and the RAND Corporation [26]. Additionally the study was funded by National Program for Health Care of Elderly, Ministry of Health and Family Welfare, Government of India, National Institute on Aging National Institutes of Health, USA and United Nations Population Fund, India [26]. Since we are interested in exploring the determinants of multi-morbidity among the older adults, we restrict our attention to the subsample of the Indian older adults and limit our sample to respondents aged 60 or above. All methods were performed in accordance with the relevant guidelines and regulations. The anthropometric measures were assessed by trained health investigators during the survey. The sample size of the present study was 31,464 respondents aged 60 years or older (male: 15,098 and female: 16,366) [26]. In case of obesity related measures, older respondents who gave consent were only considered; therefore, leading to the effective sample size for the present study to 28,050 older adults (male: 13,509 and females: 14,541). All methods were performed in accordance with the relevant guidelines and regulations and the data were fully anonymized in the study.

### Variable description

#### Outcome description

Multi-morbidity refers to the presence of two or more chronic diseases [25, 27–30] which include hypertension, chronic heart diseases, stroke, any chronic lung disease, diabetes, cancer or malignant tumour, any bone/joint disease, any neurological/psychiatric disease or high cholesterol. The variable was categorized to binary i.e., multi-morbidity (no/yes) [31–35]. The chronic diseases were assessed using the question “has any health professional ever diagnosed you with the following chronic conditions or diseases?”. The responses were available as yes and no; therefore, considered as self-reported conditions [36].

#### Explanatory variables

The variables controlled for the present study were taken into consideration after extensive literature review. Body Mass Index (BMI) was calculated as weight in kilograms divided by height in meters squared. The respondents having body mass index of 25 and above were categorized as obese/overweight [26]. Overweight/obesity was categorized as no and yes. High risk waist circumference was categorized as no and yes. Male and female who have waist circumferences of more than 102 cm and 88 cm respectively were considered as having high risk waist circumference [37]. High risk waist-hip ratio was categorized as no and yes [37]. Male and female who have waist-hip ratio of ≥ 0.90 and 0.85 cm respectively were considered as having high risk waist-hip ratio [37].

Age was categorized as young old (60–69 years), old-old (70–79 years) and oldest-old (80 + years). Education was categorized as no education/primary schooling not completed, primary completed, secondary completed and higher and above. Marital status was categorized as currently married, widowed and others (separated/never married/divorced). Working status was categorized as working, not working/retired, and never worked. Tobacco and alcohol consumption was assessed through the questions ‘have you ever smoked tobacco or used tobacco products?’, ‘And have you ever consumed alcoholic beverages such beer, wine, liquor, etc.?’. It was coded as no and yes [38]. Physical activity status was categorized as frequent (every day), rare (more than once a week, once a week, one to three times in a month) and never. The question through which physical activity was assessed was “How often do you take part in sports or vigorous activities, such as running or jogging, swimming, going to a health centre or gym, cycling, or digging with a spade or shovel, heavy lifting, chopping, farm work, fast bicycling, cycling with loads”? [26]

The monthly per capita consumption expenditure (MPCE) quintile was measured using the information related to household-level consumption of food and non-food items. The reference periods for food expenditure were seven days and for non-food expenditure were 30 days and 365 days. These expenditures have been standardized to the 30-day reference period. The MPCE is computed and used as the summary measure of consumption [26] and divided into five quintiles, i.e., from poorest to richest. Religion was categorized as Hindu, Muslim, Christian and Others. Caste was categorized as Scheduled Tribe, Scheduled Caste, Other Backward Class and others. The Scheduled Caste include a group of population which is socially segregated and financially/economically by their low status as per Hindu caste hierarchy [39]. The Scheduled Castes (SCs) and Scheduled Tribes (STs) are among the most disadvantaged socio-economic groups in India [39]. The OBC is a group of intermediate categories which are identified as “socioeconomically and educationally backward” [39]. The “other” caste category is identified as having higher social status [39]. Place of residence was categorized as rural and urban. Region was categorized as North, Central, East, Northeast, West and South.

### Statistical analysis

Descriptive statistics along with bivariate analysis was presented in the paper. The analysis was stratified by gender. To signify the association between gender differentials, proportion test [40] was used. Additionally, multivariable regression analysis [41] was used to establish the association between outcome variable (multi-morbidity) and other explanatory variables.

The binary logistic regression model is usually put into a more compact form as follows:

$$\mathrm{Logit }\left[\mathrm{P}\left(\mathrm{Y}=1\right)\right]={\beta }_{0}+\beta *X$$

The parameter ${\beta }_{0}$ estimates the log odds of the multimorbidity for the reference group, while $\beta$ estimates the maximum likelihood, the differential log odds of the multi-morbidity associated with set of predictors X, as compared to the reference group. Variance inflation factor was estimated to measure the multi-collinearity among the variable used and it was found that there was no multi-collinearity found in the variable used [42].

The multivariable analysis had four models to explain the adjusted estimates. Model-1 provides the adjusted estimates for the control variables. Model-2, model-3 and model-4 provide the interaction effects [43, 44] for overweight/obesity indicators and gender with multi-morbidity among older adults.

## Results

Table 1 presents the socio-demographic and economic profile of male and female older adults. BMI-based obesity was more prevalent among older women than men (26.3% vs. 17.6%). Similarly, a higher proportion of older women were at high-risk waist circumference and waist-hip ratio than men. A higher proportion of older women were uneducated than older men (81.4% vs. 53.1%). According to marital status, around two-fifth of older men (81%) were currently married; however, this proportion was only 44% for older women. Nearly more than half of the older women (54%) were widowed. Around 43% of the older men and 19% of women were working at the time of the survey.

**Table 1:** Socio-demographic and economic profile of older adults (*n* = 31,464), LASI, 2017–18

Background characteristics	Male		Female	
	**Sample**	**%**	**Sample**	**%**
**Obese/overweight**^**a**^				
No	11,132	82.4	10,719	73.7
Yes	2,377	17.6	3,822	26.3
**High risk waist circumference**^**a**^				
No	12,303	91.1	9,155	63.0
Yes	1,205	8.9	5,387	37.1
**High risk waist-Hip ratio**^**a**^				
No	3,318	24.6	3,146	21.6
Yes	10,184	75.4	11,402	78.4
**Age**				
Young-old	8,730	57.8	9,678	59.1
Old-old	4,702	31.1	4,803	29.4
Oldest-old	1,666	11.0	1,886	11.5
**Education**				
No education/primary not completed	8,018	53.1	13,314	81.4
Primary completed	2,235	14.8	1,297	7.9
Secondary completed	3,096	20.5	1,297	7.9
Higher and above	1,748	11.6	458	2.8
**Marital status**				
Currently married	12,242	81.1	7,211	44.1
Widowed	2,489	16.5	8,837	54.0
Others	366	2.4	318	2.0
**Work status**				
Working	6,613	43.8	3,108	19.0
Not working/Retired	7,907	52.4	5,593	34.2
Never worked	578	3.8	7,665	46.8
**MPCE quintile**				
Poorest	3,145	20.8	3,681	22.5
Poorer	3,219	21.3	3,611	22.1
Middle	3,262	21.6	3,331	20.4
Richer	2,902	19.2	3,136	19.2
Richest	2,570	17.0	2,607	15.9
**Religion**				
Hindu	12,386	82.0	13,484	82.4
Muslim	1,769	11.7	1,781	10.9
Christian	388	2.6	511	3.1
Others	555	3.7	590	3.6
**Caste**				
Scheduled Caste	2,836	18.8	3,113	19.0
Scheduled Tribe	1,166	7.7	1,389	8.5
Other Backward Class	6,925	45.9	7,308	44.7
Others	4,172	27.6	4,556	27.8
**Place of residence**				
Rural	10,879	72.1	11,322	69.2
Urban	4,219	28.0	5,044	30.8
**Region**				
North	1,863	12.3	2,096	12.8
Central	3,395	22.5	3,202	19.6
East	3,713	24.6	3,729	22.8
Northeast	437	2.9	497	3.0
West	2,457	16.3	2,941	18.0
South	3,233	21.4	3,900	23.8
**Total**	15,098	100.0	16,366	100.0

^a^The sample may differ due to missing observations; *MPCE* Monthly per capita consumption expenditure

Table 2 presents the results from the bivariate analysis of the prevalence of multi-morbidity among older adults stratified by gender. The prevalence of multi-morbidity was higher in older women with overweight/obesity than in men (44.9% vs. 38.8%), with a difference of around 6%. A similar pattern was observed for the high-risk waist-hip ratio measure. On the contrary, the prevalence of multi-morbidity was nearly 5% higher among the older men who were at a high risk of waist circumference than women. Irrespective of the age-groups, educational status, and marital status, the multi-morbidity prevalence was a bit steeper for women than men. We found that the prevalence of multi-morbidity increases with an increase in the age and the educational level for both men and women older adults. According to marital status, multi-morbidity was highly prevalent among female widows compared to male widows.

**Table 2:** Prevalence of multimorbidity among older adults stratified by gender, LASI, 2017–18, (*n* = 28,050)

Background characteristics	Male	Female	Difference	*p*-value
	**%**	**%**		
**Obese/overweight**				
No	18.3	18.5	-0.1	0.490
Yes	38.8	44.9	-6.1	0.001
**High risk waist circumference**				
No	19.6	16.1	3.4	0.001
Yes	46.3	41.2	5.1	0.030
**High risk waist-Hip ratio**				
No	14.7	24.3	-9.6	0.002
Yes	24.3	27.4	-3.2	0.001
**Age**				
Young-old	21.6	23.8	-2.3	0.001
Old-old	23.0	28.6	-5.6	0.007
Oldest-old	23.2	25.7	-2.4	0.695
**Education**				
No education/primary not completed	17.1	21.3	-4.2	0.001
Primary completed	25.4	38.1	-12.7	0.001
Secondary completed	25.9	51.5	-25.6	0.001
Higher and above	34.8	36.8	-2.0	0.008
**Marital status**				
Currently married	22.9	23.5	-0.6	0.093
Widowed	19.7	27.2	-7.5	0.001
Others	16.7	21.4	-4.7	0.174
**Work status**				
Working	14.4	12.0	2.4	0.004
Not working/Retired	28.6	24.7	3.9	0.001
Never worked	23.9	31.5	-7.6	0.001
**Tobacco consumption**				
No	26.8	26.7	0.1	0.906
Yes	19.0	21.1	-2.1	0.148
**Alcohol consumption**				
No	22.8	25.8	-2.9	0.001
Yes	20.5	13.2	7.3	0.001
**Physical activity**				
Frequent	16.4	18.0	-1.6	0.208
Rare	16.8	14.5	2.4	0.581
Never	26.0	28.0	-2.1	0.038
**MPCE quintile**				
Poorest	17.1	16.2	0.9	0.037
Poorer	19.4	21.0	-1.6	0.028
Middle	19.3	25.0	-5.7	0.001
Richer	24.5	31.0	-6.5	0.001
Richest	33.1	38.6	-5.5	0.181
**Religion**				
Hindu	21.7	24.5	-2.8	0.001
Muslim	22.7	29.2	-6.5	0.001
Christian	29.3	32.6	-3.4	0.184
Others	25.8	29.2	-3.3	0.020
**Caste**				
Scheduled Caste	18.6	20.4	-1.8	0.367
Scheduled Tribe	13.7	9.0	4.7	0.695
Other Backward Class	22.2	27.4	-5.1	0.001
Others	26.9	30.8	-3.9	0.001
**Place of residence**				
Rural	18.2	19.9	-1.7	0.023
Urban	32.5	37.8	-5.3	0.001
**Region**				
North	23.3	25.3	-2.1	0.014
Central	13.1	13.8	-0.7	0.878
East	21.1	24.7	-3.7	0.003
Northeast	18.0	14.3	3.7	0.012
West	27.9	27.6	0.3	0.058
South	28.6	35.5	-6.9	0.001
**Total**	22.2	25.4	-3.3	0.001

Difference: Male–Female; *MPCE* Monthly per capita consumption expenditure

Table 3 presents the regression estimates of multi-morbidity according to different background characteristics among older adults in India. In model-1, after controlling for other covariates, older adults with obesity/overweight were 1.6 times more likely to be multi-morbid than older adults with no obesity (AOR = 1.61; 95%CI: 1.48–1.74). Similarly, older adults with high risk waist circumference [AOR: 1.66; 95%CI: 1.52–1.80] and waist-hip ratio [AOR: 1.45; 95%CI: 1.33–1.59] also had higher odds of being multi-morbid in reference to their counterparts. The older adults in the age-group 70–79 years had 26% higher odds of having multi-morbidity than the older adults in the age-group 60–69 years (AOR = 1.26; 95%CI: 1.17–1.34). Older women had 14% lower odds of being multi-morbid than older men (AOR = 0.86; 95%CI: 0.79–0.94). The increase in the level of education was associated with higher likelihood of multi-morbidity among the elderly. Currently unmarried older adults had lower odds of having multi-morbidity than the currently married older adults (AOR = 0.81; 95%CI: 0.67–0.99). The physically inactive older adults had 33% higher odds of having multi-morbidity than frequently physically active older adults (AOR = 1.33; 95%CI: 1.21–1.46). According to MPCE quintile, older people from higher MPCE quintile had greater odds of multi-morbidity among older adults. The respondents from urban areas had 43% higher odds of being multi-morbid than their rural counterparts (AOR = 1.43; 95%CI: 1.34–1.53). The participants who belonged to the western, eastern, and southern regions had higher odds of having multi-morbidity than those of the northern region. Model-2, model-3 and model-4 represent the interaction effects. In model-3 it was found that females with high risk waist-hip ratio had 14% significantly lower likelihood to be multi-morbid than males with high risk waist-hip ratio [AOR: 0.86; 95%CI: 0.78–0.94].

**Table 3:** Logistic regression estimates for multimorbidity among older adults, LASI, 2017–18, (*n* = 28,050)

Background characteristics	Model-1	Model-2	Model-3	Model-4
	**AOR 95% CI**	**AOR 95% CI**	**AOR 95% CI**	**AOR 95% CI**
**Obese/overweight**				
No	Ref		Ref	Ref
Yes	1.61*(1.48–1.74)		1.62*(1.50–1.76)	1.60*(1.48–1.74)
**High risk waist circumference**				
No	Ref	Ref		Ref
Yes	1.66*(1.52–1.8)	1.64*(1.5–1.79)		1.66*(1.52–1.81)
**High risk waist-Hip ratio**				
No	Ref	Ref	Ref	
Yes	1.45*(1.33–1.59)	1.46*(1.34–1.6)	1.45*(1.33–1.58)	
**Age**				
Young-old	Ref	Ref	Ref	Ref
Old-old	1.26*(1.17–1.34)	1.26*(1.17–1.34)	1.26*(1.17–1.35)	1.26*(1.17–1.34)
Oldest-old	1.10(0.99–1.22)	1.10(0.99–1.22)	1.10(0.99–1.22)	1.10(0.99–1.22)
**Gender**				
Male	Ref			
Female	0.86*(0.79–0.94)			
**Education**				
No education/primary not completed	Ref	Ref	Ref	Ref
Primary completed	1.27*(1.16–1.40)	1.27*(1.16–1.39)	1.27*(1.16–1.39)	1.27*(1.16–1.4)
Secondary completed	1.29*(1.18–1.41)	1.29*(1.18–1.41)	1.29*(1.18–1.41)	1.29*(1.18–1.41)
Higher and above	1.24*(1.10–1.39)	1.24*(1.10–1.4)	1.25*(1.11–1.4)	1.24*(1.10–1.39)
**Marital status**				
Currently married	Ref	Ref	Ref	Ref
Widowed	0.97(0.90–1.04)	0.97(0.90–1.04)	0.97(0.9–1.04)	0.97(0.90–1.04)
Others	0.81*(0.67–0.99)	0.81*(0.67–0.99)	0.81*(0.67–0.98)	0.81*(0.67–0.99)
**Work status**				
Working	Ref	Ref	Ref	Ref
Not working/Retired	1.79*(1.64–1.94)	1.79*(1.64–1.94)	1.79*(1.64–1.94)	1.79*(1.64–1.94)
Never worked	1.77*(1.60–1.96)	1.76*(1.59–1.95)	1.76*(1.59–1.95)	1.77*(1.60–1.96)
**Tobacco consumption**				
No	Ref	Ref	Ref	Ref
Yes	1.01(0.94–1.09)	1.01(0.94–1.08)	1.01(0.94–1.08)	1.01(0.94–1.09)
**Alcohol consumption**				
No	Ref	Ref	Ref	Ref
Yes	1.04(0.95–1.14)	1.04(0.95–1.14)	1.04(0.95–1.14)	1.04(0.95–1.14)
**Physical activity**				
Frequent	Ref	Ref	Ref	Ref
Rare	1.03(0.91–1.16)	1.03(0.91–1.16)	1.03(0.91–1.16)	1.03(0.91–1.16)
Never	1.33*(1.21–1.46)	1.33*(1.21–1.46)	1.33*(1.21–1.46)	1.33*(1.21–1.46)
**MPCE quintile**				
Poorest	Ref	Ref	Ref	Ref
Poorer	1.23*(1.11–1.36)	1.23*(1.11–1.36)	1.23*(1.11–1.36)	1.23*(1.11–1.36)
Middle	1.39*(1.26–1.53)	1.39*(1.26–1.53)	1.38*(1.25–1.53)	1.39*(1.26–1.53)
Richer	1.60*(1.45–1.77)	1.60*(1.45–1.77)	1.60*(1.44–1.76)	1.60*(1.45–1.77)
Richest	1.98*(1.79–2.19)	1.98*(1.79–2.19)	1.98*(1.79–2.19)	1.98*(1.79–2.19)
**Religion**				
Hindu	Ref	Ref	Ref	Ref
Muslim	1.32*(1.21–1.45)	1.33*(1.21–1.45)	1.32*(1.21–1.45)	1.32*(1.21–1.45)
Christian	1.20*(1.06–1.36)	1.20*(1.06–1.36)	1.20*(1.06–1.36)	1.20*(1.06–1.36)
Others	1.12(0.97–1.29)	1.12(0.97–1.29)	1.12(0.97–1.29)	1.12(0.97–1.29)
**Caste**				
Scheduled Caste	Ref	Ref	Ref	Ref
Scheduled Tribe	0.67*(0.59–0.77)	0.67*(0.59–0.77)	0.67*(0.59–0.77)	0.67*(0.59–0.77)
Other Backward Class	1.02(0.93–1.12)	1.02(0.93–1.12)	1.02(0.93–1.12)	1.02(0.93–1.12)
Others	1.10*(1.00–1.22)	1.10*(1.00–1.22)	1.10(1.00–1.21)	1.10*(1.00–1.22)
**Place of residence**				
Rural	Ref	Ref	Ref	Ref
Urban	1.43*(1.34–1.53)	1.43*(1.33–1.52)	1.42*(1.33–1.52)	1.43*(1.34–1.53)
**Region**				
North	Ref	Ref	Ref	Ref
Central	0.71*(0.63–0.8)	0.71*(0.63–0.8)	0.71*(0.63–0.80)	0.71*(0.63–0.80)
East	1.21*(1.09–1.33)	1.21*(1.09–1.34)	1.21*(1.09–1.34)	1.21*(1.09–1.33)
Northeast	0.72*(0.63–0.83)	0.72*(0.63–0.83)	0.72*(0.63–0.83)	0.72*(0.63–0.83)
West	1.42*(1.28–1.58)	1.42*(1.28–1.58)	1.42*(1.28–1.58)	1.42*(1.28–1.58)
South	1.88*(1.71–2.07)	1.88*(1.71–2.07)	1.88*(1.71–2.07)	1.88*(1.71–2.07)
**Obese/overweight # gender**				
Yes # male		Ref		
No # male		0.66*(0.59–0.73)		
No # female		0.55*(0.49–0.62)		
Yes # female		0.93(0.82–1.06)		
**High risk waist circumference # gender**				
Yes # male			Ref	
No # male			0.72*(0.62–0.82)	
No # female			0.59*(0.50–0.69)	
Yes # female			1.03(0.90–1.20)	
**High risk waist-hip ratio # gender**				
Yes # male				Ref
No # male				0.67*(0.6–0.76)
No # female				0.60*(0.53–0.69)
Yes # female				0.86*(0.78–0.94)

*Ref* Reference; #: Interaction; *if *p* < 0.05; *AOR* Adjusted odds ratio, MPCE, Monthly per capita consumption expenditure

## Discussion

The present study based on large country-representative survey information of older Indian adults aged 60 years and above, has shown the associations between several anthropometric measures and multi-morbidity in older ages with a special focus on gender difference in such associations. Although bivariate analyses in the current study showed female disadvantage in the prevalence of multi-morbidity, higher likelihood of multi-morbidity among older men than women in the multivariate analysis after adjusting for potential confounders was contrary to earlier studies in developed as well as developing countries that have revealed greater odds of multi-morbidity among older women, relating to their longer life expectancy and poor health status compared to older men [33, 45–48]. Since there is a dearth of studies due to lack of availability of large-scale data on multi-morbidity in low and middle income countries, the present study adds to the scientific evidence in geriatric research.

Older adults who were measured as obese in the study population, consistent with a couple of previous studies, were 61% more likely to report having multi-morbidity than older adults with no obesity [14, 22, 47]. Similarly, other two measures of obesity in our study, waist circumference and waist-to-hip ratio which are seldom studied in Indian context were also significantly associated with a higher prevalence of multi-morbidity among both older men and women, indicating that obesity is an important risk factor for morbidity in older ages. Other population-based studies conducted in low and middle income countries also found relationships between multi-morbidity and measures of waist circumference and waist-to-hip ratio in older ages [22, 49]. The finding is in agreement with a recent study in India that found that adults aged 45 and above with obesity and high-risk waist circumference or waist-hip ratio were more likely to develop cardiovascular diseases than their counterparts [50], and another study reporting that a higher BMI among general population is associated with a greater prevalence of chronic diseases [51].

The current analysis shows that oldest age group is at reduced risk for multi-morbidity and is consistent with earlier studies [52, 53]. This might be an effect of survival bias in this cross-sectional analysis with increasing number of chronic diseases seems to be associated with mortality [52]. Similarly, it has been revealed that older age is associated with less accurate self-reporting of diseases [54], which may also explain the lower odds of multi-morbidity in oldest age group in our study. Considering the socioeconomic associations of multi-morbidity, the current results are in parallel with studies that have shown a different pattern in developing countries compared to developed ones with a greater prevalence among people with higher socioeconomic circumstances [55–57]. The study found higher prevalence of multi-morbidity among individuals with higher levels of education, belonging to households with higher wealth quintiles and non-SC/STs. This also supports the positive relationship between wealth and health gradient in low and middle income countries shown by higher multi-morbidity burden among higher socioeconomic groups [58]. This however can be attributed to the higher rate of surveillance bias in the diagnosis of chronic diseases among socioeconomically vulnerable populations [57]. It is also accompanied by their lack of information on the need for diagnosis and treatment of the diseases [14]. Again, as evidence suggests it is oftentimes difficult to obtain appropriate medical advices on different combinations of chronic diseases suffered by older individuals especially in poor socioeconomic settings [59]. Thus, health interventions should pay special attention on in detecting and treating multi-morbid older populations and frame disease-specific policies accordingly. Future studies with multiple disease combinations are also warranted for better understanding the morbidity pattern since clusters of diseases and their frequencies could inform treatment guidelines on how healthcare can be designed and delivered [60, 61]. Research on the dynamic changes in these combinations by analysing longitudinal data (possibly with future waves of LASI survey) is also required to understand the ageing trajectories of multi-morbidity.

In agreement with a few cross-sectional as well as longitudinal studies, in the present study, late-life physical inactivity was associated with a higher likelihood of multi-morbidity [2, 62–64]. The finding supports the previous evidence suggesting that the changes in lifestyle and increased sedentary behaviour among older adults are associated with increased rates of multi-morbidity [25, 65]. This is also an important finding with crucial impact in terms of preventive strategies that calls for special attention from health-decision makers in the country. Nevertheless, results on lifestyle factors such as smoking and alcohol consumption with no significant association with multi-morbidity are inconsistent with multiple studies that have shown tobacco use and alcohol drinking as major risk factors of higher prevalence of multiple chronic conditions [66–68]. Contrarily, some studies found that daily or weekly consumption of alcohol was inversely associated with multi-morbidity [69]. Thus, the current finding with no significance may be attributed to the dichotomous nature of the response which captures only ever use of tobacco and alcohol, suggesting the need for further investigation.

Furthermore, the interactive effect of gender in the associations of obesity-related measures and multi-morbidity shows that older men with overweight/obesity are at greater risk for multi-morbidity than their women counterparts. The male disadvantage in being multi-morbid observed in the current analyses can be attributed to the hormonal differences between males and females, genetic factors and differences in clinical severity [70].. Similarly, as documented, men may have a higher number of chronic conditions that are clinically more severe than among women, this may partially explain the higher prevalence of multi-morbidity among older men [70].

Finally, the regional variations and urban- rural gradient in the prevalence of multi-morbidity suggest that older adults from southern states of the country which are socioeconomically advantaged with relatively developed healthcare system and higher levels of education and income [58], and those from urban areas were at increased risk for being multi-morbid. This can be explained by the greater prevalence of several diseases in more urbanised areas and in wealthy people due to their lower engagements in physical activity and farming and unhealthy dietary habits [71, 72]. The finding can also be explained by the variations in healthcare facilities and differential accessibility which result in higher rate of disease diagnosis in urban and better-off regions of the country.

There were some limitations to be acknowledged in the present study. The cross-sectionality of the study makes it unfeasible to infer the causation in the observed directions of the relationships. Also, it uses a relatively simple definition of counting diseases for measuring multi-morbidity in variance with earlier studies [33, 73, 74]. The chronic conditions selected in our study of multi-morbidity did not include skin conditions, eye diseases, thyroid, urinary problems, liver diseases and gastro-intestinal problems, thus, future investigation is required with a higher number of diseases. Previous studies also suggested that the prevalence of multi-morbidity varies according to data source and multiple data can provide better understanding of the disease prevalence [27]. Additionally, along with a few missing cases in the data that lead to possible selection bias, the self-reported chronic conditions in our study are subject to measurement error due to under-diagnosis. Again, with regard to the tobacco and alcohol consumption as factors of multi-morbidity, the dose and duration and the consumption pattern were not considered in the study due to unavailability of data. Despite these limitations, there are major strengths too. The study utilizes the information of large nationally representative sample of the older population and obesity-related indicators that are measured. Hence, the findings of the current study are generalizable to the older adults in India and other aging populations in low and middle income countries.

## Conclusion

The results of the study show significant gender differences in the prevalence of multi-morbidity between older men and women, men being at increased risk in the multivariate analysis which is uncommon in the existing epidemiological research. The findings highlight the need for better management of chronic conditions in older adults in primary care, increased prevention measures including nutritional interventions, physical activity promotion etc., information and physician education with a special focus on those who are obese, overweight or with high-risk waist circumference and waist-hip ratio. Interactive effect of male gender with anthropometric measures on multi-morbidity reported in our study probably due to increased unhealthy behaviours among men, requires further research. Also, studies are required on multi-morbidity patterns and several combinations of morbidities and its impact stratified by gender among the aging population in India.

## Data Availability

The study utilizes a secondary source of data that is freely available in public domain through request from https://iipsindia.ac.in/sites/default/files/LASI_DataRequestForm_0.pdf

## Abbreviations

AOR: Adjusted odds ratio
MPCE: Monthly per capita expenditure
CI: Confidence interval
BMI: Body mass index
LASI: Longitudinal Aging Study in India
WC: Waist circumference
WHR: Waist hip ratio
